# Altered Recruitment of the Attention Network Is Associated with Disability and Cognitive Impairment in Pediatric Patients with Acquired Brain Injury

**DOI:** 10.1155/2015/104282

**Published:** 2015-09-10

**Authors:** Sandra Strazzer, Maria A. Rocca, Erika Molteni, Ermelinda De Meo, Monica Recla, Paola Valsasina, Filippo Arrigoni, Susanna Galbiati, Alessandra Bardoni, Massimo Filippi

**Affiliations:** ^1^Acquired Brain Injury Unit, Scientific Institute “Eugenio Medea”, Via Don Luigi Monza 20, 23842 Bosisio Parini, Italy; ^2^Neuroimaging Research Unit, Institute of Experimental Neurology, San Raffaele Scientific Institute, Vita-Salute San Raffaele University, Via Olgettina 60, 20132 Milan, Italy; ^3^Department of Neurology, San Raffaele Scientific Institute, Vita-Salute San Raffaele University, Via Olgettina 60, 20132 Milan, Italy

## Abstract

We assessed abnormalities of brain functional magnetic resonance imaging (fMRI) activity during a sustained attention task (Conners' Continuous Performance Test (CCPT)) in 20 right-handed pediatric acquired brain injury (ABI) patients versus 7 right-handed age-matched healthy controls, and we estimated the correlation of such abnormalities with clinical and cognitive deficits. Patients underwent the Wechsler Intelligence Scale for Children (WISC), Wisconsin Card Sorting Test, and Functional Independence Measure (FIM) evaluations. During fMRI, patients and controls activated regions of the attention network. Compared to controls, ABI patients experienced a decreased average fMRI recruitment of the left cerebellum and a decreased deactivation of the left anterior cingulate cortex. With increasing task demand, compared to controls, ABI patients had an impaired ability to increase the recruitment of several posterior regions of the attention network. They also experienced a greater activation of frontal regions, which was correlated with worse performance on FIM, WISC, and fMRI CCPT. Such abnormal brain recruitment was significantly influenced by the type of lesion (focal versus diffuse axonal injury) and time elapsed from the event. Pediatric ABI patients experienced an inability to optimize attention network recruitment, especially when task difficulty was increased, which likely contributes to their clinical and cognitive deficits.

## 1. Introduction

Acquired brain injury (ABI) is one of the most common causes of childhood disability. Children with severe ABI suffer from debilitating cognitive and functional deficits [1], which may delay skill acquisition and impair peer interaction [2]. The basic and superior components of attention [3] are usually affected following ABI. Some studies also detected inattentive behaviors, including distractibility, inability to inhibit responses to irrelevant information, and tendency to overprocess redundant stimuli [4]. Impairment of attentive and inhibitory functions can persist well beyond the acute phase of injury [5].

Functional magnetic resonance imaging (fMRI) abnormalities associated with cognitive deficits following ABI have not yet been characterized univocally. Using working memory tasks [6–10], the majority of fMRI studies of adult and pediatric patients with different types of ABI, such as traumatic brain injury (TBI), have provided conflicting results, especially for moderate “load” conditions (i.e., for conditions with a moderate cognitive demand) [6, 8, 10]. However, these studies are concordant in highlighting a failure of TBI patients to increase the recruitment of the working memory circuitry with increasing task difficulty [6–10]. Such a failure has been interpreted as a lack of processing reserve in TBI patients who recruit all their cognitive capacity to perform moderate load tasks and have little or no reserve for increased loads [8].

Conners' Continuous Performance Test (CCPT) [11] is commonly used to assess attention deficits since it measures the ability to maintain attention for critical but temporally infrequent events presented in the absence of simultaneous distracters. Such a test has been successfully used to investigate attention in healthy adults [12] and in patients with psychiatric and neurologic diseases [13, 14]. Only a seminal study of five TBI children with a normal attentive performance has used CCPT [3] and found an overactivation of brain regions involved in sustained attention, mainly located in the frontal and parietal lobes.

In this study, we applied fMRI during CCPT performance to investigate the recruitment of the sustained attention system with increasing task demand and to assess its relationship with cognitive performance and clinical disability in a relatively large group of pediatric ABI patients. Our working hypothesis was that an abnormal recruitment of this network with increasing task difficulty should have been associated with worse clinical and neuropsychological outcomes. To gain additional insight into the factors likely to be associated with fMRI abnormalities, we also investigated the effect of the type of lesions (i.e., focal lesions versus diffuse axonal injury (DAI)) and time elapsed from the event to the performance of this experiment.

## 2. Materials and Methods

### 2.1. Ethics Committee Approval

Approval was received from the local ethical standards committee on human experimentation of the Scientific Institute “E. Medea” (Bosisio Parini, Italy), and written informed consent was obtained from all parents/caregivers prior to study enrolment.

### 2.2. Subjects

Twenty pediatric patients (mean age = 14.0 years, range = 7.5–18 years, 11 females) were recruited. Patients' inclusion criteria were (1) a diagnosis of ABI based on anamnestic, clinical, and neuroimaging data; (2) an age at insult between 6 and 18 years; (3) no residual attentive deficits (to avoid bias due to different task performances between patients and controls during fMRI acquisition), as assessed by the computerized CCPT [15]; (4) ability to undergo neuropsychological testing and follow simple motor instructions, such as clicking on a mouse button with the right index finger; and (5) right-handedness. The exclusion criteria were (1) congenital neurological pathology (determined from medical records or relatives' reports); (2) history of social or educational disadvantage preceding injury (according to the Four-Factor Index of Social Status) [16]; (3) behavioral disability before injury; (4) motor disability such as severe tetraparesis; and (5) inability to undergo MRI (e.g., metal implants or claustrophobia).

As a control group, seven right-handed age-matched healthy pediatric volunteers (mean age = 12.8 years, range = 7.4–17.7 years, 6 females), with no previous history of neurologic dysfunction and a normal neurologic examination, were recruited consecutively among families of the personnel involved in the study and by word of mouth.

### 2.3. Clinical and Neuropsychological Assessment

Within one week from the fMRI examination, all patients underwent clinical and neuropsychological assessment. Neuropsychological evaluation was performed by an experienced neuropsychologist unaware of MRI results, using a standardized battery of tests, appropriate for age, which included the Wechsler Intelligence Scale for Children-third edition (WISC-III) [17] for intelligence and the Wisconsin Card Sorting Test (WCST) [18] for executive functions. Eighteen patients also underwent the “Child Behavior Checklist 4/18” to assess problematic behaviors and emotional disorders [19] (scores >60 are considered pathological on the Internalizing, Externalizing, and Total Problem Scales; scores >70 are considered pathological on all other scales).

The patients' medical history was collected by an experienced neurologist, including the Glasgow Coma Scale (GCS) score (normal value = 15) at insult [20] and the number of days of coma. The Functional Independence Measure (FIM) Scale (value in healthy controls older than 97 months = 126) [21] (which measures patient's mobility, cognitive capabilities, and daily living independence) and the Disability Rating Scale (DRS) (normal value = 0) [22] (which quantifies awareness and responsivity, dependence on caregivers, and psychosocial adaptability) were administered.

### 2.4. fMRI Experimental Design

The computerized version of the CCPT [23] was implemented with the Presentation software (http://www.neuro-bs.com/, version 14.8). Stimuli consisted of alphabetical letters, presented in pseudo-randomized sequence, one at a time, at the centre of the MRI screen. Letters were black on a white background. The stimuli were presented through MRI goggles and a four-button ergonomic response pad was secured to the patients' right hand through Velcro strips. Subjects were given standardized instructions to respond as fast as possible whenever a letter other than X appeared and to withhold the response when the letter X was shown. The total amount of presented stimuli was 252, and every stimulus remained on the screen for 500 ms (stimulus duration). The probability of the infrequent stimulus (X) was set at 14.3% (*n* = 36). The test was administered in 6 stimulation blocks, lasting 98 sec each. Each block consisted of three subblocks, in which stimuli were presented in random order with different interstimulus intervals (ISI) (fixed at 1, 2, and 4 sec, resp.). Blocks were interleaved by rest periods, during which a meaningless image (randomly oriented geometrical lines) was shown to the subjects for 20 sec. Since our patients did not have residual attentive deficits, we hypothesized that task difficulty increased from ISI-4 to ISI-1 condition, because of the increasing response speed required from the subject. The total protocol duration was 11.8 min. All subjects were trained to perform the task before MRI investigation. Percentages of correct and incorrect responses as well as reaction times (RT) were recorded.

### 2.5. MRI Acquisition

Using a 3.0 T scanner (Philips Medical Systems, Best, Netherlands), the following brain MRI scans were acquired from all subjects: (i) T2^*∗*^-weighted single-shot echo-planar imaging sequence during the CCPT task (repetition time [TR]/echo time [TE] = 2000/30 ms; flip angle = 85°; field of view = 240 mm^2^; matrix = 128 × 128; slice thickness = 4 mm; 354 sets of 30 contiguous axial sections) and (ii) a 3D T1-weighted fast-field-echo sequence (TR/TE = 25/4.6 ms; flip angle = 30°; 200 contiguous axial sections; voxel size = 0.9 × 0.9 × 0.8 mm; matrix size = 256 × 256; field of view = 230 mm^2^). All subjects enrolled were able to complete fMRI acquisition without interruptions.

### 2.6. fMRI Analysis

fMRI data were analyzed using the SPM8 software (http://www.fil.ion.ucl.ac.uk/spm/software/spm8/). Prior to statistical analysis, all images were realigned to the first one, normalized to a custom-made pediatric template, created from 3D T1-weighted scans using a pipeline optimized for processing data from children populations and implemented in the Template-O-Matic toolbox [24], and smoothed with a 10 mm Gaussian kernel. The mean cumulative translations were 0.24 mm (SD = 0.31 mm) for controls and 0.37 mm (SD = 0.33 mm) for ABI patients (*p* = 0.4) and the mean rotations were <0.3 degrees in both groups (*p* = 0.5). Changes in blood oxygenation level dependent contrast associated with the performance of the CCPT task were assessed on a voxel by voxel basis, using the general linear model and the theory of Gaussian fields [25]. A first-level design matrix, including motion parameters as regressors, was built and specific effects were tested by applying appropriate linear contrasts (activation and deactivation). For each subject, the three task conditions (ISI-1, ISI-2, and ISI-4) were contrasted with the rest condition. Areas showing increased/decreased activation with increasing task difficulty were identified by creating a linear contrast (load effect) from ISI-4 to ISI-1. Peaks of fMRI activity were localized using the Automatic Anatomical Labelling toolbox [26] in the Montreal Neurological Institute (MNI) standard space. A schematic representation of brain regions involved in this study is shown in Supplementary Figure 2 (see Supplementary Materials available online at http://dx.doi.org/10.1155/2015/104282).

### 2.7. Statistical Analysis

CCPT performance was compared between groups using a two-sample *t*-test, adjusted for age and sex. A second-level analysis with SPM8 was performed to assess (1) the average fMRI activation and deactivation during the CCPT (i.e., average activation of the ISI-1, ISI-2, and ISI-4 conditions) and the load effect in controls and ABI patients, separately, and in female and male ABI patients, separately (one-sample *t*-test); (2) the comparisons of average and load fMRI activation between controls and patients (two-sample *t*-test, age and sex adjusted) and between female and male ABI patients (two-sample *t*-test); (3) the comparisons of average and load fMRI activation between patients with focal lesions only versus patients with DAI, as well as patients in the chronic (time ≥ 1 year) versus those in the acute/subacute phase of injury (acute: time < 6 months; subacute: 6 months ≤ time < 1 year; these two phases were combined since only 3 patients were in a subacute phase) (full factorial model, age and sex adjusted); (4) the correlation between fMRI activation and clinical and neuropsychological scores (multiple regression models). Conjunction analysis [27] was performed to assess fMRI abnormalities of a given patients' group versus the others included in the full factorial models. Results were tested both at *p* < 0.001 uncorrected and at *p* < 0.05, familywise error (FWE) corrected.

## 3. Results

### 3.1. Clinical, Neuropsychological, and Neuroimaging Assessment

Table 1 summarizes the main demographic, clinical, and neuroimaging features of ABI patients. Supplementary Figure 1 shows imaging findings on anatomical 3D T1-weighted scans at the time of fMRI acquisition in individual patients.

Mean age at insult was 12.1 years (range = 7.2–17.8 years), median days of coma were 10.5 (range = 0–105), median GCS score at insult was 7 (range = 3–15), median FIM score was 122.5 (range = 78–126), and median DRS score was 2.5 (range = 0–5). Etiology was TBI in 15 patients (75%), hemorrhagic stroke (due to arteriovenous malformation) in 3 (15%), ischemic stroke in 1 (5%), and brain tumor (treated with surgery) in 1 (5%). Among the 15 TBI patients, 6 experienced an impact due to acceleration and 8 due to deceleration (for one patient this was not possible to be defined) [28]. The type of ABI, lesion location, and presence of DAI were assessed on imaging done at the time of injury: 7 TBI patients had a closed injury and 8 a penetrating injury; 13 experienced DAI [29]. Eleven (55%) patients were in an acute/subacute stage and 9 (45%) in a chronic condition.

All ABI patients were assessed using the WISC-III (Supplementary Table 1). Their mean verbal intelligence quotient (VIQ) was 82.5 (SD = 23.9), with 7 patients performing below the normality range (NR = 100.0 ± 25.0); mean performance IQ (PIQ) was 78.2 (SD = 19.8), with 8 patients performing below the NR; and mean final score IQ (FSIQ) was 77.9 (SD = 22.4), with 10 patients performing below the NR. Six patients were unable to complete the WCST, due to defeatist approach to the test (*n* = 4) or inability to fulfil the task entirely (*n* = 2). The individual performance of the remaining patients is reported in Supplementary Table 1. Only one of the 18 patients tested for depression had abnormal scores at the “Child Behavior Checklist 4/18” (data not shown).

### 3.2. CCPT fMRI Task Performance

During fMRI, CCPT performance (percentage of correct and incorrect responses, RT) did not differ significantly between ABI patients and healthy controls (Supplementary Table 2).

### 3.3. CCPT Task-Related Activation/Deactivation

#### 3.3.1. Within-Group Analysis

**Figure 1:**
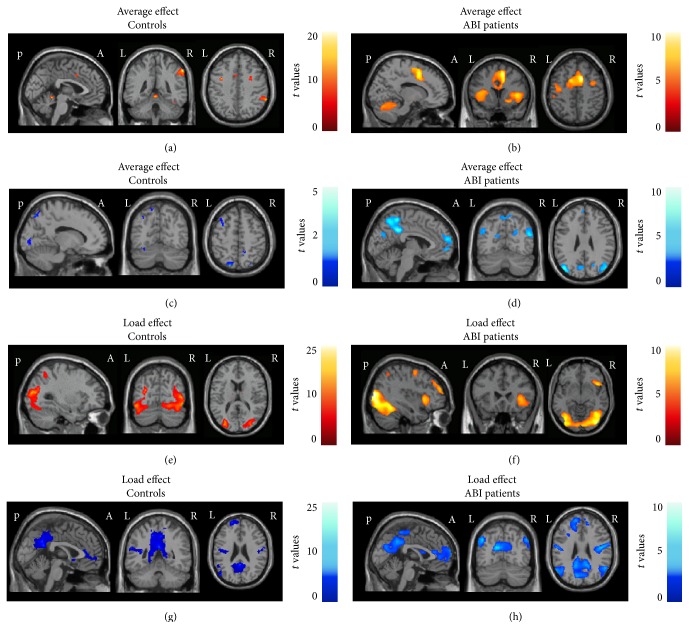
Brain regions showing significant activation (colour coded with red-yellow scales) and deactivation (colour coded with blue-light blue scales) during fMRI with the modified Conners Continuous Performance Test (CCPT) (average condition and load effect) in healthy controls and patients with acquired brain injury (ABI). (a) and (c) Average activation and deactivation in healthy controls; (b) and (d) average activation and deactivation in ABI patients; (e) and (g) activation and deactivation during the load effect in healthy controls; (f) and (h) activation and deactivation during the load effect in ABI patients. Results are shown at *p* < 0.001, uncorrected. Images are in neurological convention. See text for further details.

Table 2 and Figure 1 report brain regions significantly activated/deactivated during the CCPT task in controls and ABI patients. Both groups showed task-related activation in regions of the frontal, parietal, and temporal lobes, cerebellum, insulae, and left thalamus (Figures 1(a) and 1(b)). In controls, deactivation was observed in the left fusiform gyrus, middle occipital gyrus (MOG), precuneus, and inferior frontal gyrus (IFG) (Figure 1(c)). ABI patients experienced a more distributed pattern of deactivation, which also included the right superior temporal gyrus (STG), left superior frontal gyrus (SFG), and right middle frontal gyrus (MFG) (Figure 1(d)). With increasing task demand (load effect), both groups showed a linear increase of recruitment of the bilateral occipital lobes, lingual gyri, cerebellum, and left inferior parietal lobule (IPL) (Figures 1(e) and 1(f)). ABI patients also recruited the precentral gyrus and supplementary motor area (SMA), bilaterally (Figure 1(f)). Significant deactivation was observed in both groups in the precuneus/posterior cingulate cortex (PCC), STG, SFG, MFG, and insula, bilaterally (Figures 1(g) and 1(h)). The results obtained from the analysis of TBI patients only were virtually similar to those reported for the whole group of ABI patients (data not shown). No significant gender-related differences of fMRI recruitment were found in ABI patients.

#### 3.3.2. Between-Group Comparisons

**Figure 2:**
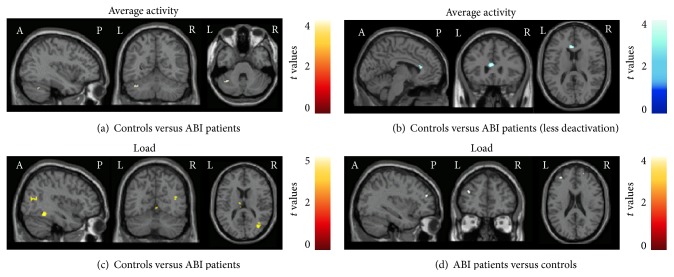
Brain regions showing significantly different fMRI activation during the modified Conners Continuous Performance Test (CCPT) between controls and patients with acquired brain injury (ABI). (a) and (b) Between-group differences during the average condition; (c) and (d) between-group differences during the load effect. Differences of activation are coded in red-yellow scales, whereas differences of deactivation are coded in blue-light blue scales. Results are shown at *p* < 0.001, uncorrected. Images are in neurological convention. See text for further details.

Compared to controls, ABI patients showed decreased fMRI recruitment of the left cerebellum, crus I (MNI coordinates: −36, −56, −32, *t* value = 4.12) (Figure 2(a)) and a decreased deactivation of the left anterior cingulate cortex (ACC) (MNI coordinates: −4, −28, 16, *t* value = 4.27) (Figure 2(b)). During the load condition, compared to controls, ABI patients experienced reduced activation of the right MOG (MNI coordinates: 38, −72, 14, *t* value = 4.03), right fusiform gyrus (MNI coordinates: 40, −50, −14, *t* value = 4.63), left thalamus (MNI coordinates: −8, −14, 14, *t* value = 5.58), right cerebellum, lobule VI (MNI coordinates: 32, −76, 20, *t* value = 3.95), and vermis (MNI coordinates: 0, −62, −2,  *t* value = 3.64) (Figure 2(c)). They also experienced an increased activation of the left MFG (MNI coordinates: −34, 46, 22, *t* value = 3.86) and right SFG (MNI coordinates: 22, 56, 24, *t* value = 3.69) (Figure 2(d)). Similar results were obtained when comparing TBI patients only with controls.

#### 3.3.3. Lesion and Time Effects

With increasing task difficulty (load effect), compared to controls and patients with focal lesions (*n* = 7), DAI patients (*n* = 13) experienced an increased recruitment of the left SFG (MNI coordinates: −26, 36, 30; *k* = 23; conjunction analysis: *p* < 0.001). Compared to the other two groups, patients with focal lesions showed an increased recruitment of the right postcentral gyrus (MNI coordinates: 44, −24, 38; *k* = 40; conjunction analysis: *p* < 0.001) and left STG (MNI coordinates: −46, −44, 14; *k* = 30; conjunction analysis: *p* < 0.001).

**Figure 3:**
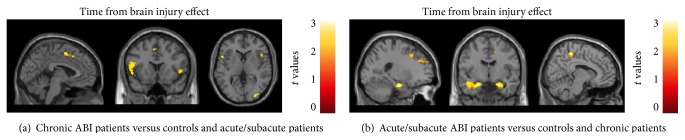
Brain regions showing significant group differences of fMRI activation during the modified Conners Continuous Performance Test (CCPT) (load effect) between acute/subacute and chronic patients with acquired brain injury (ABI) and healthy controls. (a) Significantly increased activation in chronic patients versus controls and acute/subacute patients; (b) significantly increased activation in acute/subacute patients versus controls and chronic patients. Results are shown at *p* < 0.001, uncorrected. Images are in neurological convention.

With increasing task demand, compared to acute/subacute patients and controls, chronic patients showed an increased activation (conjunction analysis: *p* < 0.001) of the left SMA (MNI coordinates: −8, 4, 50; *k* = 181), right (MNI coordinates: 48, 18, 2; *k* = 69) and left (MNI coordinates: −48, 18, 14; *k* = 211) IFG, and right inferior occipital gyrus (IOG) (MNI coordinates: 48, −78, −8; *k* = 139). Compared to the other two groups, acute/subacute patients showed an increased activation (conjunction analysis: *p* < 0.001) of the left (MNI coordinates: −18, 34, 30; *k* = 132) and the right (MNI coordinates: 18, 64, 24; *k* = 26) SFG, right (MNI coordinates: 26, −20, −18; *k* = 277) and left (MNI coordinates: −22, −10, −22; *k* = 697) hippocampal/parahippocampal gyrus, and right precuneus (MNI coordinates: 8, −40, 56; *k* = 119) (Figure 3).

#### 3.3.4. Analysis of Correlations

**Figure 4:**
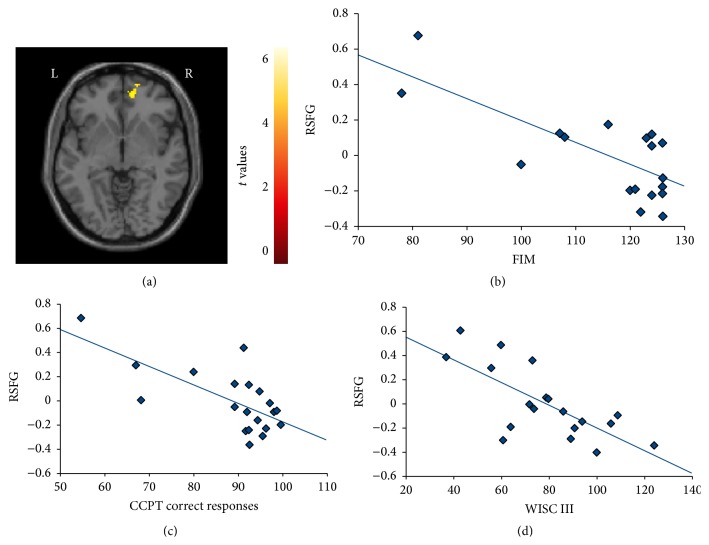
Correlations (*p* < 0.001) between increased functional magnetic resonance imaging activation of the right superior frontal gyrus (SFG) (a) during the load fMRI condition of the Conners Continuous Performance Test (CCPT) in acquired brain injury patients versus (b) Functional Independent Measure (FIM) scores; (c) the percentage of correct responses during CCPT; and (d) Wechsler Intelligence Scale for Children (WISC-III) score.

In ABI patients, significant (*p* < 0.001) correlations were found between the following:

(i) decreased activation of the left cerebellum and worse WISC-III (PIQ) score (*r* = 0.67) and longer RT at the CCPT (*r* = −0.71);
(ii) decreased deactivation of the left ACC and worse FIM scores (*r* = −0.78);
(iii) increased activation of the right SFG during the load condition and worse scores at FIM (*r* = −0.73), WISC-III (FSIQ) (*r* = −0.72), WISC-III (PIQ) (*r* = −0.71), and WISC-III (VIQ) (*r* = −0.69), as well as with a lower percentage of correct responses (*r* = −0.69) at CCPT (Figure 4);
(iv) decreased activation of the right vermis during the load condition and worse FIM scores (*r* = 0.70);
(v) decreased activation of the right MOG (*r* = −0.71) and right fusiform gyrus (*r* = −0.66) during the load condition and longer RT at CCPT.

## 4. Discussion

Pediatric ABI patients usually experience deficits of physical and cognitive functions which persist beyond the acute phase of injury. Given that abnormalities of the brain attention network have been suggested to contribute to long-term sequelae of ABI, we used fMRI to investigate the recruitment of such a network during the performance of a sustained task of attention and inhibition in a relatively large group of ABI patients. We also assessed the relationship of fMRI abnormalities with patients' clinical and neuropsychological findings. In addition to the investigation of the average pattern of activation of the attention network and the topographical distribution of abnormalities, we also performed a parametric analysis to quantify the ability of the network to modulate its activation with increasing task demand. To avoid bias in the interpretation of the findings related to different task performance between patients and controls, we selected patients without attention deficits, as assessed using the clinical CPT (which is not affected by behavioral ratings and practice bias) [30] during study enrolment. The recruitment of patients without attention deficits allowed us to investigate neural abnormalities due to compensative reorganization after injury and ensured their capability to sustain attentional load.

During the CCPT task, both healthy controls and ABI patients experienced a distributed pattern of activation of the attention network, which included regions located in the occipital, temporal, and parietal lobes and cerebellum. The two groups also showed a consistent deactivation of areas which are considered nodes of the default mode network [31], including the precuneus/PCC, STG, and medial SFG. Compared to controls, ABI patients showed a significantly lower recruitment of the left cerebellum (crus I) and a reduced deactivation of the ACC. The cerebellum, in particular the neocerebellar crus I/II, is involved in information updating, salience detection, abstract reasoning, and response selection [32]. Activation of cerebellar crus I has been shown in adults and adolescents during inhibitory control in Go/No-Go tasks [33]. As a consequence, the reduced cerebellar activation we observed in patients is likely due to deficits in executive control. The ACC is known to be deactivated when an attention to external stimuli is needed [34]. Such a deactivation is likely to reflect the recruitment of inhibitive resources for halting processes interfering with a correct performance of the task. Therefore, the impaired ACC deactivation we found in ABI patients might be the reflection of a decreased monitoring of cognitive conflicts, error detection, and immediate response readjustments secondary to the dysfunction of the subserving neural structures. However, we cannot rule out the fact that this finding could be the consequence of the use of a different strategy to perform the task. Our results conflict with those of a previous study of 5 TBI patients [3], which found increased activation of regions mainly located in the frontal and parietal lobes in patients when compared to controls. Several factors might contribute to explain this discrepancy, including differences in CPT task setup (use of only one difficulty level in the previous study versus parametric task in ours), the criterion used to select healthy subjects (who had had orthopaedic injury in the previous study), the number of patients and their clinical and demographic characteristics (5 chronic TBI patients in the previous study), and the method used for statistical analysis (including the use of a more liberal threshold in the previous study).

The most intriguing results of our study are those derived from the parametric analysis of attention network activation, which demonstrated an impaired ability of ABI patients to increase the recruitment of several regions of the network (mainly located in the occipital lobes, cerebellum, and thalamus) with increasing task demand. These findings are in agreement with previous fMRI studies of the working memory network in adult and pediatric TBI patients [6–10], pointing towards a global impairment of functional reserve (not specifically limited to a given cognitive network) in patients suffering of this condition. Such an impaired functional reserve exerts an impact on the clinical outcome of these patients, since it was correlated with a worse score at FIM and longer RT at CCPT. Importantly, during the load condition, compared to controls, ABI patients also experienced an increased recruitment of the right SFG. This is likely to reflect the result of maladaptation, since it was correlated to a worse performance at FIM, WISC, and fMRI CCPT. At present, the role of the right prefrontal cortex in mediating cognitive performance is still debated. Indeed, previous studies in TBI and ABI yielded inconsistent and sometimes conflicting results [35, 36]. Many factors are likely to contribute to discrepancies among studies, including clinical characteristics of the patients enrolled (e.g., severity of trauma, time occurring from the event), measures of performance analyzed (e.g., accuracy versus RT), and additional variables possibly related to task performance and fMRI activity (e.g., type of lesion). Among the studies which analyzed the correlation between behavioral performance and fMRI activity during attentive or working memory tasks, several authors [7, 37, 38] found a negative correlation between increased recruitment of the right dorsolateral prefrontal cortex (including the SFG) and poor task performance, suggesting that an increased activity of frontal lobe might reflect inefficient utilization of neuronal resources.

We also explored the effects on our findings of type of lesions and time elapsed from onset of injury. Consistently with previous pathological [39] and diffusion tensor MRI [40] studies which have shown that DAI hits frontal nodes of the cognitive networks, we found that recruitment of frontal lobe areas was more pronounced in patients with DAI than in those with focal brain lesions. Attention network recruitment also varied significantly according to the stage of the disease (acute/subacute versus chronic), with a more pronounced recruitment of regions which are part of the default mode network (SFG, hippocampus, and precuneus) in the early phases after the insult. This agrees with results of a recent longitudinal resting state fMRI study which has shown dynamic modifications of functional connectivity between the hippocampi and frontal circuitry in patients with ABI [41].

Our study is not without limitations. First, the number of healthy controls was relatively small when compared to ABI patients. Nevertheless, it should be considered that the enrolment of healthy pediatric controls is a challenging task, due to relatives' reluctance to give their consent. Second, although the number of patients included was relatively large, the study subgroups (stratified on sex, type and location/severity of lesions, or time elapsed between the event and MRI assessment) were relatively small, thus not allowing us to perform powered subanalyses. Similarly, we could not assess the influence of depressive disorders, since only one of our patients had such a disturbance. Third, to increase subjects' compliance, we did not acquire diffusion tensor MRI at the time of fMRI acquisition, which would have provided important pieces of information on the architectural integrity of the attention network and its influence on both fMRI and neuropsychological findings. Finally, the study is cross-sectional, thus preventing the assessment of the dynamic relationship between abnormalities of functional activation and clinical outcomes.

## Supplementary Material

The Supplementary material includes the list of abbreviations used in the paper, two Tables (reporting the results of the neuropsychological and behavioural assessments performed on study subjects) and two Figures (the first one showing conventional MRI images of the pediatric patients involved in this study, and the second one showing a schematic representation of brain regions activated by the functional MRI task).

## Figures and Tables

**Table 1:** Main demographic, clinical, and magnetic resonance imaging characteristics of patients with acquired brain injury (ABI).

Number	Sex	Age at event (years)	Age at study (years)	Cause	Dynamics of impact^*∗*^	Type of ABI^*∗*^	Diffuse axonal injury^*∗*^	Lesion location^*∗*^	Time after trauma	Days of coma	Glasgow Coma Scale (GCS)	Functional Independence Measure (FIM)	Disability Rating Scale (DRS)
1	M	7.2	7.5	Hemorrhagic stroke	n.a.	n.a.	0	Left temporoparietal arteriovenous malformation	A	8	8	81	5
2	M	9.2	10.0	Brain trauma	1	1	1	Diffuse axonal injury	S	25	7	107	5
3	F	11.0	13.1	Brain trauma	2	2	1	Diffuse axonal injury	C	30	4	122	1
4	F	11.6	12.5	Brain trauma	1	1	0	Cerebral and cerebellar atrophy; left temporoparietal cortical contusion; paramedian bilateral frontal contusions	S	21	6	108	4
5	F	15.6	15.8	Hemorrhagic stroke	n.a.	n.a.	0	Left frontoinsular arteriovenous malformation	A	0	12	124	3
6	M	16.5	16.5	Brain trauma	n.e.	n.e.	1	Diffuse axonal injury	A	5	8	121	3
7	M	7.9	9.5	Brain trauma	2	2	1	Diffuse axonal injury; left frontal and right temporoparietal small contusions	C	9	4	116	4
8	F	8.2	15.2	Brain trauma	1	1	1	Diffuse axonal injury	C	24	3	126	2
9	M	13.1	13.4	Brain trauma	1	2	1	Diffuse axonal injury	A	0	15	120	2
10	F	8.9	9.0	Brain trauma	1	2	1	Diffuse axonal injury, frontotemporal contusion	A	4	7	126	0
11	F	14.1	16.8	Brain trauma	2	2	0	Small right frontal and temporal contusions	C	35	5	126	1
12	F	16.2	16.3	Hemorrhagic stroke	n.a.	n.a.	0	Left frontoinsular hematoma left subdural hematoma	A	1	10	124	2
13	M	13.5	14.4	Brain trauma	2	2	1	Diffuse axonal injury; left cerebellar and temporobasal cortical contusions	S	8	8	126	3
14	F	14.6	18.0	Brain trauma	2	1	1	Diffuse axonal injury	C	12	5	123	0
15	F	9.7	12.6	Ischemic stroke	n.a.	n.a.	0	Left insular and basal ganglia regions	C	0	15	100	4
16	F	11.6	14.8	Brain tumor	n.a.	n.a.	0	Posterior fossa ependymoma	C	105	7	78	5
17	F	10.1	12.8	Brain trauma	1	2	1	Diffuse axonal injury	C	20	6	126	0
18	M	17.8	18.0	Brain trauma	2	1	1	Diffuse axonal injury	A	20	7	124	0
19	M	10.7	15.7	Brain trauma	2	1	1	Diffuse axonal injury	C	23	5	121	3
20	M	14.0	18.0	Brain trauma	2	1	1	Diffuse axonal injury	C	5	8	124	0

^*∗*^Evaluated on MRI scans performed during the acute stage (within 6 months) of disease, which also included a gradient echo scan in the majority of cases.

n.e. = not evaluable; n.a. = not applicable; M = male; F = female.

Dynamics of impact: acceleration = 1; deceleration = 2. A traumatic injury due to acceleration occurs when the head accelerates because of the impact (e.g., a person hit by a car); a traumatic injury due to deceleration occurs when the head decelerates because of the impact (e.g., a person falling to the ground).

Diffuse axonal injury: absent = 0; present = 1.

Type of acquired brain injury (ABI): closed = 1; penetrating = 2. An open brain injury occurs when an object penetrates the skull and enters the brain; a closed brain injury occurs when there is a force on the head that leaves the skull intact but results in injury to the brain tissue.

Time after trauma: acute = A, if time <6 months, subacute = S if 6 months ≤ time < 12 months, and chronic = C, if time ≥12 months.

**Table 2:** Brain regions significantly activated/deactivated during fMRI with the modified Conners Continuous Performance Test (CCPT) task (average and load conditions) in healthy controls and patients with acquired brain injury (ABI) (one-sample *t*-test, *p* < 0.001).

Group	Contrast	Brain regions	Side	BA	MNI space coordinates	Cluster extent *k*	*T* values
*Activation*							

Healthy controls	Average activation	SPL	R	40	62 −44 48	174	20.0^*∗*^
		SMA	L	6/24	−34 2 42	52	13.1
		MFG	R	10/44/48	50 16 20	107	8.2
		MFG	L	46	−38 36 32	48	5.5
		IFG	R	44/48	34 20 36	518	8.2^*∗*^
		MTG	L	37	−48 −66 6	10	8.0
		Vermis IV-V	—	—	2 −46 −8	50	17.8
		Cerebellum, crus I	L	—	−44 −62 −28	22	7.0
		Thalamus	L	—	−8 −7 13	22	4.1
		Insula	R	48	42 15 −4	174	5.79

Healthy controls	Load activation	Calcarine cortex/ITG	R	17/37/19	14 −88 0	4114	29.3^*∗*^
		FFG/MOG	L	19	−46 −64 −16	2636	25.5^*∗*^
		MFG	R	46/47	44 56 −4	113	7.9
		IPL	L	7	−32 −56 50	79	11.1
		Insula	R	48	34 24 8	27	3.37
		Lingual gyrus	R	27	4 −32 −2	46	7.3
		Cerebellum, crus II	L	—	−6 −84 −36	66	9.2

ABI patients	Average activation	SPL	R	40	52 −48 40	71	4.3
		SMA	L/R	6	12 10 52	3946	10.5^*∗*^
		MFG	R	46	36 38 28	610	6.8^*∗*^
		IFG/putamen	R	47/48	40 26 6	2309	7.7^*∗*^
		MTG/STG	L/R	21/42/48	50 −48 4	381	5.4
		Vermis IV-V-VI/	—	—	0 −48 −10	2792	10.6^*∗*^
		Cerebellum, crus I	L		−36 −64 −32		
		Thalamus	L	—	−18 −14 12	552	6.9
		Insula	L	48	−34 14 12	1262	6.0^*∗*^

ABI patients	Load activation	MOG	R	18/19	34 −86 10	2070	11.0^*∗*^
		MOG	L	18/19	−28 −92 8	1267	9.8^*∗*^
		IPL	L	7	−28 −56 48	214	6.0
		SMA	L	6	−6 8 50	1081	8.0^*∗*^
		Precentral gyrus	L	6	−44 −6 52	1661	7.2^*∗*^
		MFG	R	10/46	36 50 22	488	6.2^*∗*^
		Insula	R	47	36 14 10	24	4.4
		Cerebellum	L	—	−34 −76 −22	500	8.8^*∗*^

*Deactivation*							

Healthy controls	Average activation	MOG	L	19	−30 −80 28	860	8.1^*∗*^
		Precuneus	L	5	−8 −46 58	16	3.9
		Fusiform gyrus	L	37	−28 −34 −22	627	5.6
		Orbital IFG	L	47	−50 40 −2	120	5.0

Healthy controls	Load activation	Precuneus	L/R	30	12 −46 46	2171	21.7^*∗*^
		SFG	L	10	−16 60 20	1229	27.8^*∗*^
		PCC	—	30	12 −52 16	256	15.9^*∗*^
		STG	R	48	44 −12 2	150	7.9^*∗*^
		MTG	L	21	−48 −68 20	298	6.5^*∗*^
		Insula	L	48	−36 −18 4	38	6.6

ABI patients	Average activation	SFG	L	10	−6 38 54	74	4.0
		Precuneus/MCC	L	7	−8 −60 54	6602	7.8^*∗*^
		STG/MTG	R	22	60 −4 10	206	4.2
		MOG	L	18	−30 −92 4	513	4.8^*∗*^
		MFG	L/R	10	8 60 −2	485	6.0^*∗*^

ABI patients	Load activation	STG	R	48	46 −12 6	2195	15.4^*∗*^
		Precuneus	L/R	30	−10 −42 58	1519	11.3^*∗*^
		Postcentral gyrus	L	48	−52 −10 18	667	11.1^*∗*^
		SFG	L/R	8/10/11	−20 16 −14	426	3.6
		PCC	—	—	−10 −40 38	261	7.5^*∗*^
		Insula	L	48	−26 12 −14	428	3.6

R = right; L = left; BA = Brodmann Area; FFG = fusiform gyrus; IFG = inferior frontal gyrus; IOG = inferior occipital gyrus; MOG = middle occipital gyrus; IPL = inferior parietal lobule; SPL = superior parietal lobule; MFG = middle frontal gyrus; MOG = middle occipital gyrus; ITG = inferior temporal gyrus; MTG = middle temporal gyrus; SMA = supplementary motor area; STG = superior temporal gyrus; MCC = middle cingulate cortex; PCC = posterior cingulate cortex.

^*∗*^ *p* < 0.05, familywise error (FWE) corrected for multiple comparisons.

## Abbreviations

ABI: Acquired brain injury
ACC: Anterior cingulate cortex
CCPT: Conners' Continuous Performance Test
DAI: Diffuse axonal injury
DRS: Disability Rating Scale
FIM: Functional Independence Measure
fMRI: Functional magnetic resonance imaging
FSIQ: Final score intelligence quotient
FWE: Familywise error
GCS: Glasgow Coma Scale
IFG: Inferior frontal gyrus
IOG: Inferior occipital gyrus
IPL: Inferior parietal lobule
ISI: Interstimulus interval
MFG: Middle frontal gyrus
MNI: Montreal Neurological Institute
MOG: Middle occipital gyrus
NR: Normality range
PCC: Posterior cingulate cortex
PIQ: Performance intelligence quotient
RT: Reaction time
SFG: Superior frontal gyrus
SMA: Supplementary motor area
STG: Superior temporal gyrus
TBI: Traumatic brain injury
TE: Echo time
TR: Repetition time
VIQ: Verbal intelligence quotient
WCST: Wisconsin Card Sorting Test
WISC-III: Wechsler Intelligence Scale for Children-third edition.
