# Supplementary material for: Altered Recruitment of the Attention Network Is Associated with Disability and Cognitive Impairment in Pediatric Patients with Acquired Brain Injury
Source: Neural Plast. 2015 Sep 10;2015:104282. doi: 10.1155/2015/104282 (PMC4581560; doi:10.1155/2015/104282)
Supplement: Supplementary file 1 — The Supplementary material includes the list of abbreviations used in the paper, two Tables (reporting the results of the neuropsychological and behavioural assessments performed on study subjects) and two Figures (the first one showing conventional MRI images of the pediatric patients involved in this study, and the second one showing a schematic representation of brain regions activated by the functional MRI task). [file 104282.f1.pdf]

Supplementary Table 1. Results of the neuropsychological assessment by Wechsler Intelligence Scale III, Wisconsin Card Sorting Test and in-scanner Conners' Continuous Performance Test in patients with acquired brain injury (ABI).

N°	Wechsler Intelligence Scale III			Conners' Continuous Performance Test			Wisconsin Card Sorting Test (raw scores)					
	VIQ	PIQ	FSIQ	Correct responses (%)	Incorrect responses (%)	Reaction time (ms)	Correct responses	Perseverative responses	Perseverative errors	Non-perseverative errors	Total Errors	Categories
1	56	94	72	55	45	454.57	64	77	78	95	83	2
2	82	72	74	68	32	562.33	35	55	55	117	67	0
3	90	93	91	94	6	370.39	n.e.	n.e.	n.e.	n.e.	n.e.	n.e.
4	65	65	60	80	20	566.18	86	102	105	81	91	4
5	48	51	43	91	9	384.88	81	86	83	88	85	4
6	77	59	64	92	8	600.60	84	25	22	22	44	5
7	81	72	73	92	8	465.58	80	88	89	94	91	4
8	47	41	37	67	33	389.65	82	91	92	81	85	1
9	99	76	86	92	8	399.36	82	95	94	83	87	5
10	92	97	94	98	2	436.26	n.e.	n.e.	n.e.	n.e.	n.e.	n.e.
11	102	77	89	100	0	511.32	n.e.	n.e.	n.e.	n.e.	n.e.	n.e.
12	58	73	61	92	8	455.56	65	121	120	105	112	6
13	88	75	79	97	3	381.15	78	101	99	97	99	6
14	103	115	109	92	8	337.24	n.e.	n.e.	n.e.	n.e.	n.e.	n.e.
15	48	75	56	89	11	438.02	78	107	107	123	117	6
16	74	52	60	96	4	654.35	86	94	93	93	94	4
17	86	79	80	95	5	343.46	81	100	97	82	89	4
18	128	114	124	99	1	516.68	69	119	118	122	121	6
19	126	85	106	96	4	502.67	n.e.	n.e.	n.e.	n.e.	n.e.	n.e.
20	100	99	100	89	11	247.69	n.e.	n.e.	n.e.	n.e.	n.e.	n.e.

Abbreviations: n.e.= not executed; VIQ=Verbal Intelligence Quotient; PIQ=Performance Intelligence Quotient; FSIQ=Full Scale (or Total) Intelligence Quotient.

Supplementary Table 2. Behavioral results during functional magnetic resonance imaging Conners' Continuous Performance Test (CCPT) from healthy controls and patients with acquired brain injury (ABI).

	Healthy controls	ABI patients	p values*
CCPT correct responses [%] (SD)	86.6 (9.9)	88.8 (12.0)	0.62
CCPT incorrect responses [%] (SD)	13.4 (9.9)	11.2 (12.0)	0.62
CCPT mean reaction time [ms] (SD)	437 (90)	451 (100)	0.61

Abbreviations: M=male; F=female; SD=standard deviation.

*Two-sample t test.

Supplementary Figure 1 Legend. Individual 3D T1-weighted fast-field-echo sequence from the 20 pediatric patients with acquired brain injury (ABI). Three axial sections are depicted for each patient, in neurological convention (right is right). From left to right, sections provide a view of the midbrain and cerebellum, deep gray matter nuclei, and cortical structures. Patients' numbers match the cases listed in Table 1. Whenever visible, focal damage has been highlighted by red arrows.

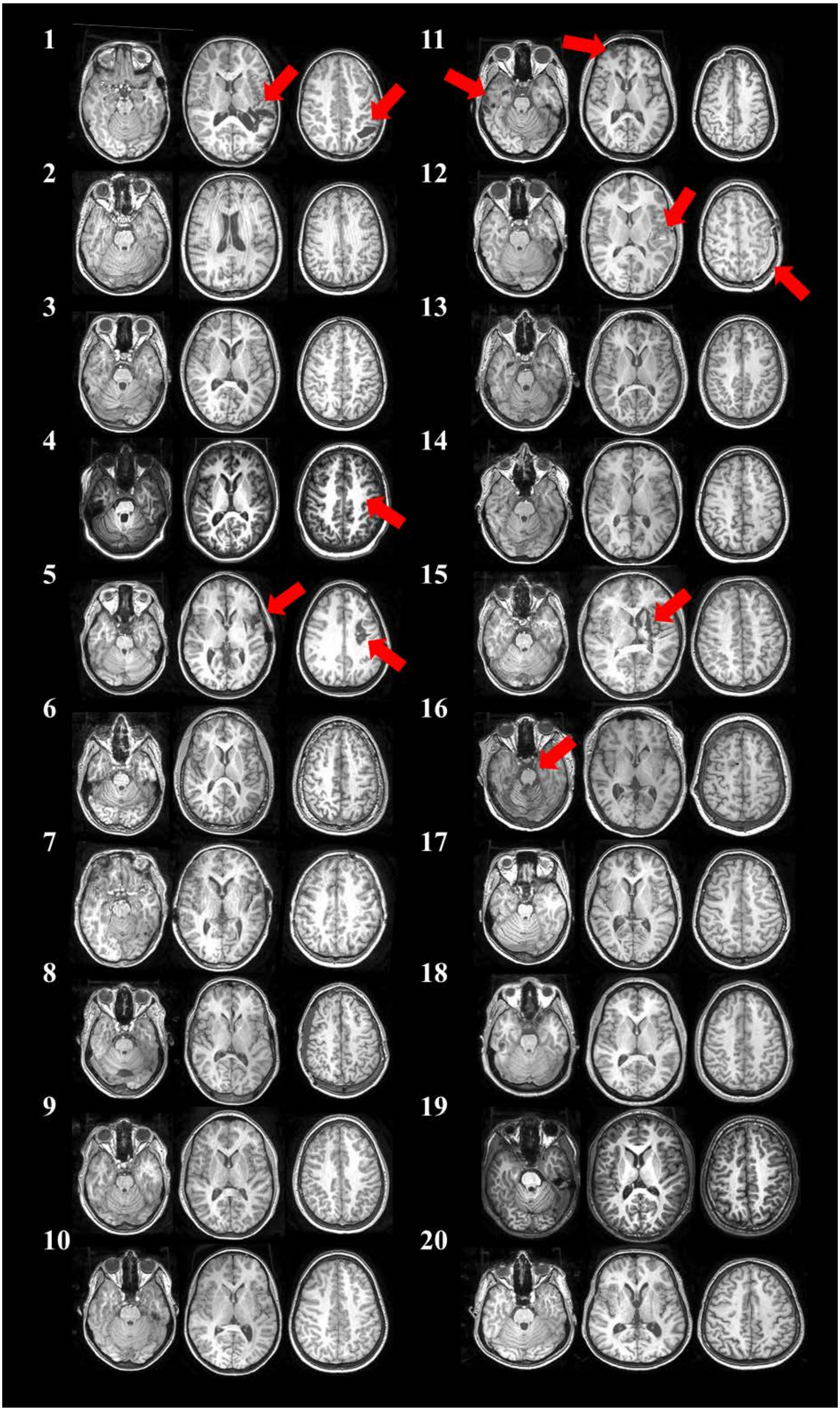
1
2
3
4
5
6
7
8
9
10
11
12
13
14
15
16
17
18
19
20
